# The association of genetic variants in *FGFR2* with osteoporosis susceptibility in Chinese Han population

**DOI:** 10.1042/BSR20190275

**Published:** 2019-06-04

**Authors:** Yang Yang, Mengxue Fei, Xinying Zhou, Yuejun Li, Dadi Jin

**Affiliations:** 1 Department of Orthopedics, Academy of Orthopedics of Guangdong Province, The Third Affiliated Hospital, Southern Medical University, Guangzhou, China; 2 Department of Environmental Health, School of Public Health, Guangxi Medical University, Nanning, China

**Keywords:** FGFR2, Haplotype, Osteoporosis, Polymorphisms

## Abstract

**Objective:** The present study was conducted for exploring the influence of fibroblast growth factor 2 receptor (*FGFR2*) gene polymorphisms on osteoporosis occurrence risk in the Chinese population.

**Methods:** Polymerase chain reaction–restriction fragment length polymorphism (PCR–RFLP) was conducted for the genotyping of polymorphism in 145 osteoporosis patients and 123 controls. The status of Hardy–Weinberg equilibrium was detected in the control group. Genotype and allele frequency comparison of polymorphism between the two groups was performed by χ^2^ test, odds ratio (OR) with 95% confidence interval (95% CI) was used for the result expression about the association of *FGFR2* polymorphisms with osteoporosis. Furthermore, the results were adjusted by clinical features via logistic regression analysis.

**Results:** AA genotype and A allele of rs2420946 were significantly associated with the increased risk of osteoporosis development adjusted by clinical features (OR = 2.238, 95% CI = 1.055–4.746; OR = 1.482, 95% CI = 1.042–2.019). Similarly, CC genotype and C allele frequencies of rs1219648 were detected the significant difference between the case and control groups (*P*<0.01); moreover, it was still significant by the adjustion of clinical features, which indicated that rs1219648 was significantly associated with the risk of osteoporosis occurrence (OR = 2.900, 95% CI = 1.341–6.271; OR = 1.602, 95% CI = 1.126–2.279). Haplotype T-A-C-T also obviously increased the occurrence risk of osteoporosis (OR = 1.844, 95% CI = 1.180–2.884). Besides, the significant interaction of *FGFR2* polymorphisms with drinking status in osteoporosis was also found (*P*<0.05), especially rs2981579.

**Conclusion:** *FGFR2* rs2420946 and rs1219648 polymorphisms may be the risk factor of osteoporosis in Chinese population. Furthermore, the interaction of *FGFR2* polymorphisms with drinking may play an important role in osteoporosis etiology.

## Introduction

Osteoporosis is a common systemic bone disease in elderly characterized by the progressive bone mass loss and the degeneration of bone microarchitecture, which results in the decreased bone strength and increased risk of fracture [1,2]. The prevalence of osteoporosis increases with the age [3] and is also increasing annually. According to epidemiological investigation, its incidence rate is 14.94% in 2008 and the percentage reach to 27.96% during 2012–2015 in China [4]. The occurrence of osteoporosis threatens or damages the physical health and life quality of patients, also causing enormous economic losses [5]. Revealing the pathogenesis of osteoporosis is an urgent problem so as to prevent and treat it. Osteoporosis is a multifactorial disease influenced by genetic and environmental factors [6,7], of which genetic factors can account for 60–85% of the influence [8]. Although a number of genetic factors have been reported to be associated with osteoporosis, these are not enough.

Fibroblast growth factor 2 receptor (FGFR2) is a kind of receptor tyrosine kinase (RTK) belonging to immunogloblin superfamily [9]. It is mainly secreted by epithelial cells and mesenchymal cells and plays the important role in skeletal and gland development, limbs and skin formation as well as various organs formation, combined with FGFs, including FGF1-4, FGF6, 7 and so on [10,11]. As a RTK in membrane surface, tissue and spatio-temporal specific expression of FGFR2 greatly affects FGFs/FGFR2 biological function [12,13]. In previous studies, FGFR2 has been reported to be associated with osteoblasts development. Enhanced FGFR2 signaling can promote immaturate osteoblasts hyperplasia, inhibit its differentiation and lead to apoptosis [14]. The balance of bone formation regulated osteoblasts and bone absorption regulated by osteoclasts is the mechanism of osteoporosis. Recently, FGFR2 is found to regulate chondrocyte development too [15]. However, the role of *FGFR2* in osteoporosis is rarely studied nowadays.

In the present study, we explored the influence of *FGFR2* on osteoporosis via genetic variants based on the Chinese Han population and four most common single nucleotide polymorphisms (SNPs) in *FGFR2* were included in the present study. Moreover, the interaction of *FGFR2* SNPs in osteoporosis development was also analyzed.

## Materials and methods

### Subjects

In the present study, a total of 268 subjects were enrolled from the Third Affiliated Hospital, Southern Medical University during September, 2017 and December, 2018, including 145 confirmed osteoporosis and 123 healthy controls as the case and control groups, respectively. Osteoporosis patients were diagnosed by specialist physicians through measuring the bone mineral density (BMD) of lumbar vertebra and bilateral femoral proximal femur, tibia using dual X-ray absorption-metry (DXA), according to the diagnosis criteria of World Health Organization (WHO) [16], that was, the T-score of BMD measured by DXA was less than or equal to −2.5 S.D. Patients would be excluded who suffered from hyperthyroidism, diabetes, liver and renal failure, other diseases influencing bone metabolism. In addition, none the individuals had taken antiosteoporosis drugs before sampling. The case group consisted of 62 males and 83 females from 47 to 83 years old. BMD of the controls was normal and they were also not subject to diseases associated with bone metabolism, including 47 males and 76 females with the age range of 44–82 years old. The controls were frequency-matched with the cases in gender and age. All subjects were Chinese Han population without any blood relationship. The present study was supported by the Ethics Committee of the Third Affiliated Hospital, Southern Medical University and the subjects had been informed the objective. Certainly, written consents were signed by every subject in the present study.

In the meanwhile, the basic characteristics of subjects were investigated and recorded by trained doctor in an Excel form using the uniform questionnaire, including age, gender, body mass index (BMI), cigarette and alcohol consumption.

### Sample collection

Each subject was drew 2-ml peripheral venous blood in the early morning and put it into given blood collection tube with EDTA2Na anticoagulation, then stored at −80°C for genomic DNA collection.

### Genotyping

Whole blood genomic DNA of subject was extracted by Blood Genome DNA Extraction Kit (TaKaRa, Dalian) according to the manufacturer’s instruction, −20°C storage for standby application. Polymerase chain reaction–restriction fragment length polymorphism (PCR–RFLP) was used to conduct the genotyping of *FGFR2* polymorphisms. PCR primers were synthesized in Invitrogen, Shanghai according to the sequences reported by Siddiqui and Liu [17,18]. The detailed sequences are shown in Table 1. The 25 μl PCR system was used in the present study and PCR procedure was as follows: 95°C pre-degeneration for 5 min, followed by 35 cycles of 95°C degeneration for 30 s, annealing at 55–60°C for 30 s, 72°C extension for 30 s, and final extension at 72°C for 10 min. PCR products were detected by 1.0% agarose gel electrophoresis (AGE). Then eligible PCR products were digested by restriction enzymes (Table 1) and enzyme-digested products were separated by 3% AGE, then observed on the Gel Doc 2000 system (Bio-Rad, U.S.A.).

**Table 1:** PCR primers sequences of *FGFR2* polymorphisms

Polymorphism		Primer sequences (5′-3′)	Restriction enzyme
rs2981582	For.	CGTGAGCCAAGCCTCTACTT	*Aci* I
	Rev.	TAAGTGTGCTGTTCATTCA	
rs1219648	For.	ATGGTACCGGTTTCCCAA	*BspQ* I
	Rev.	TGTGATTTGTATGTGGTAG	
rs2981579	For.	GTGACTCCCTTCATCGTG	*Pst* I
	Rev.	GGCTCCTGGTCTATTTCTC	
rs2420946	For.	GTGGAAAGGGACGAAGTT	*Hinp1* I
	Rev.	TTGGTGGAAGAGTCAGAAGA	

### Statistics analysis

The genotype and allele frequencies of polymorphisms in *FGFR2* were gained by counting. The genotype distribution of polymorphism in the control group was checked whether complied with Hardy–Weinberg equilibrium (HWE). Genotype and allele frequencies of *FGFR2* polymorphisms were compared between the case and control groups by χ^2^ test, odds ratio (OR) and 95% confidence interval (CI) were used to express the risk intensity of osteoporosis resulted from the genetic variants of *FGFR2*. The results were adjusted by basic characteristics of subjects through logistic regression analysis. The above operation was completed by SPSS 18.0 software. The linkage disequilibrium of polymorphisms in the present study was also analyzed by Haploview software. The *P*<0.05 was defined as the statistically significant difference.

## Results

### The demographic characteristics of subjects

The clinical basic characteristics of subjects in the case and control groups were showed in Table 2. The mean age of the cases and controls was respective 65.63 ± 9.31 and 64.15 ± 8.77 years old. The ratio of males and females in the case group was 1.34, the ratio in the control group was 1.62. No significant difference between the two groups was detected in age and gender (*P*>0.05). We did not detect the significant association between smoking and osteoporosis occurrence (*P*=0.243). However, the mean value of BMI between the two groups was significantly different (24.80 ± 2.11 & 24.26 ± 1.95, *P*=0.030). Nearly one third of the cases were drinkers, but the percentage was only 22% in the controls and the significant difference was found between the two groups (*P*=0.043). So BMI and alcohol consumption were the independent influence factors of osteoporosis.

**Table 2:** The basic characteristics of subjects

Characteristic		Case, *n*=145	Control, *n*=123	*P*-value
Age (age)	The range	47–83	44–82	
	Mean age	65.63 ± 9.31	64.15 ± 8.77	0.184
Gender (%)	Male	62 (42.76)	47 (38.21)	0.450
	Female	83 (57.24)	76 (61.79)	
BMI (kg/m^2^)	Mean value	24.80 ± 2.11	24.26 ± 1.95	0.030
Smoking (%)	Yes	37 (25.52)	24 (19.51)	0.243
	No	108 (74.48)	99 (80.49)	
Drinking (%)	Yes	48 (33.10)	27 (21.95)	0.043
	No	97 (66.90)	96 (78.05)	

### The genotype and allele distribution of *FGFR2* polymorphisms in the case and control groups

The genotype and allele frequencies of *FGFR2* polymorphisms are shown in Table 3. First, all of four polymorphisms in the study were consistent with HWE in the genotype distribution of the control group (*P*>0.05), which suggested that this population derived from the same Mendelian population. For rs2981582, we detected the significant difference in TT genotype and T allele frequencies between the case and control groups (*P*=0.021, 0.016); however, the difference became not significant adjusted by clinical features (*P*>0.05). Both of AA genotype and A allele frequencies in rs2420946 were significantly higher in osteoporosis patients than that in the controls, respectively compared with GG genotype and G allele (*P*=0.027, 0.025). Adjusted by clinical features, the differences still remain significant (*P*<0.05), which indicated rs2420946 was associated with the significantly elevated risk of osteoporosis occurrence (AA vs GG: OR = 2.238, 95% CI = 1.055–4.746; AA+AG vs GG: OR = 1.796, 95% CI = 1.048–3.079; A vs G: OR = 1.482, 95% CI = 1.042–2.109). We also detected the significant difference between the two groups in CC genotype and C allele frequencies of rs1219648 (*P*=0.007, 0.009), the significant difference was still detected adjusted by clinical features (*P*=0.007, 0.009), so rs1219648 was also a risk factor of osteoporosis development (CC vs TT: OR = 2.900, 95% CI = 1.341–6.271; CC vs T+CT: OR = 2.106, 95% CI = 1.082–4.099; CC+CT vs TT: OR = 1.870, 95% CI = 1.070–3.265; C vs T: OR = 1.602, 95% CI = 1.126–2.279). But there was no any significant difference between the case and control groups in genotypes or alleles of rs2981579 (*P*>0.05), so were results adjusted by clinical features.

**Table 3:** The genotype and allele frequencies comparison of *FGFR2* polymorphisms between the case and control groups

Genotype/allele		Case, *n*=145 (%)	Control, *n*=123 (%)	OR (95% CI)	*P*-value	OR^a^ (95% CI)	*P*-value^a^	*P*_HWE_
rs2981582	CC	51 (35.17)	58 (47.15)	Ref.	-			0.983
	CT	68 (46.90)	53 (43.09)	1.459 (0.867–2.455)	0.154	1.345 (0.790–2.288)	0.275	
	TT	26 (17.93)	12 (9.76)	2.464 (1.129–5.379)	0.021	2.085 (0.936–4.644)	0.072	
	TT vs CC+CT	119 (82.07)	111 (90.24)	2.021 (0.973–4.199)	0.056	1.780 (0.841–3.767)	0.131	
	TT+CT vs CC	94 (64.83)	65 (52.85)	1.645 (1.006–2.688)	0.047	1.482 (0.895–2.453)	0.126	
	C	170 (58.62)	169 (68.70)	Ref.	-			
	T	120 (41.38)	77 (31.30)	1.549 (1.084–2.213)	0.016	1.419 (0.984–2.045)	0.061	
rs2420946	GG	36 (24.83)	45 (36.59)	Ref.	-			0.609
	AG	78 (53.79)	61 (49.59)	1.598 (0.921–2.775)	0.095	1.664 (0.944–2.934)	0.078	
	AA	31 (21.38)	17 (13.82)	2.279 (1.092–4.759)	0.027	2.238 (1.055–4.746)	0.036	
	AA vs GG+AG	114 (78.62)	106 (86.18)	1.696 (0.887–3.241)	0.108	1.635 (0.841–3.179)	0.147	
	AA+AG vs. GG	109 (75.17)	78 (63.41)	1.747 (1.032–2.956)	0.037	1.796 (1.048–3.079)	0.033	
	G	150 (51.72)	151 (61.38)	Ref.	-			
	A	140 (48.28)	95 (38.62)	1.484 (1.051–2.094)	0.025	1.482 (1.042–2.109)	0.029	
rs1219648	TT	32 (22.07)	42 (34.15)	Ref.	-			0.238
	CT	79 (54.48)	65 (52.84)	1.595 (0.907–2.807)	0.104	1.619 (0.905–2.897)	0.105	
	CC	34 (23.45)	16 (13.01)	2.789 (1.316–5.916)	0.007	2.900 (1.341–6.271)	0.007	
	CC vs TT+CT	111 (70.35)	107 (86.99)	2.048 (1.068–3.927)	0.029	2.106 (1.082–4.099)	0.028	
	CC+CT vs. TT	113 (77.93)	81 (65.85)	1.831 (1.066–3.146)	0.028	1.870 (1.070–3.265)	0.028	
	T	143 (49.31)	149 (60.57)	Ref.	-			
	C	147 (50.69)	97 (39.43)	1.579 (1.119–2.228)	0.009	1.602 (1.126–2.279)	0.009	
rs2981579	CC	27 (18.62)	35 (28.46)	Ref.	-			0.965
	CT	80 (55.17)	61 (49.59)	1.700 (0.931–3.106)	0.083	1.807 (0.875–3.730)	0.110	
	TT	38 (26.21)	27 (21.95)	1.824 (0.903–3.688)	0.093	1.638 (0.880–3.050)	0.120	
	TT vs CC+CT	107 (73.79)	96 (78.05)	1.263 (0.718–2.222)	0.418	1.282 (0.717–2.292)	0.401	
	TT+CT vs CC	118 (81.83)	88 (71.54)	1.738 (0.980-3.083)	0.057	1.690 (0.936–3.051)	0.082	
	C	134 (46.21)	131 (53.25)	Ref.	-			
	T	156 (53.79)	115 (46.75)	1.326 (0.943–1.864)	0.104	1.317 (0.929–1.867)	0.123	

Note: a: OR and *P* value was adjusted by clinical parameters; HWE: Hardy-Weinberg equilibrium

### The linkage disequilibrium analysis of *FGFR2* polymorphisms in osteoporosis development

In the present study, we investigated the interaction of *FGFR2* polymorphisms in the onset of osteoporosis through linkage disequilibrium analysis and the results showed the strong linkage disequilibrium among *FGFR2* rs2981582, rs2420946, rs1219648 and rs2981579 polymorphisms. We detected a total of five haplotypes in this Chinese population, namely C-G-T-C, T-A-C-T, C-A-C-T, T-G-T-C and C-G-T-T haplotypes. The frequency of haplotypes is shown in Table 4. Only T-A-C-T haplotype showed the significant association with osteoporosis occurrence (OR = 1.844, 95% CI = 1.180–2.884, *P*=0.007).

**Table 4:** The haplotype analysis of *FGFR2* polymorphisms in osteoporosis occurrence

Site1-site2-site3-site4	Haplotype (%)		OR (95% CI)	*P*-value
	Case, 2n = 290	Control, 2n = 246		
C-G-T-C	90 (31.03)	104 (42.28)	Ref.	
T-A-C-T	83 (28.62)	52 (21.14)	1.844 (1.180–2.884)	0.007
C-A-C-T	57 (19.66)	43 (17.48)	1.532 (0.942–2.491)	0.085
T-G-T-C	37 (12.76)	25 (10.16)	1.710 (0.957–3.056)	0.069
C-G-T-T	16 (5.52)	20 (8.13)	0.924 (0.452–1.890)	0.830

Note: site1: rs2981582; site2: rs2420946; site3: rs1219648; site4: rs2981579

### The interaction of *FGFR2* polymorphisms and drinking status in osteoporosis risk

The relationship between the interaction of *FGFR2* polymorphisms with drinking status and osteoporosis risk was also explored and the results were showed in Table 5. We found that drinkers with CT+TT genotype of rs2981582 had the significantly higher risk of osteoporosis occurrence than non-drinkers with CC genotype (*P*=0.008, OR = 2.693, 95% CI = 1.287–5.634). moreover, we gained the similar results in rs2420946 (*P*=0.004, OR = 3.133, 95% CI = 1.432–6.852) and rs1219648 (*P*=0.002, OR = 3.141, 95% CI = 1.524–7.648). Drinkers with any genotype of rs2981579 and non-drinkers with CT+TT genotype all significantly increased the risk of osteoporosis occurrence compared with non-drinkers with CC genotype (*P*=0.009, OR = 4.488, 95% CI = 1.409–14.298; *P*=0.003, OR = 3.452, 95% CI = 1.496–7.968; *P*=0.008, OR = 2.566, 95% CI = 1.256–5.243).

**Table 5:** The interaction analysis of *FGFR2* polymorphisms and drinking status in osteoporosis risk

	Genotype	Drinking	Case, *n*=145	Control, *n*=123	*P*-value	OR (95% CI)
rs2981582	CC	No	36	47		
	CC	Yes	15	11	0.202	1.780 (0.730–4.339)
	CT+TT	No	61	49	0.097	1.625 (0.915–2.886)
	CT+TT	Yes	33	16	0.008	2.693 (1.287–5.634)
rs2420946	GG	No	23	35		
	GG	Yes	13	10	0.168	1.978 (0.744–5.260)
	AG+AA	No	74	61	0.053	1.846 (0.987–3.452)
	AG+AA	Yes	35	17	0.004	3.133 (1.432–6.852)
rs1219648	TT	No	21	31		
	TT	Yes	11	11	0.445	1.476 (0.542–4.023)
	CT+CC	No	76	65	0.096	1.726 (0.905–3.291)
	CT+CC	Yes	37	16	0.002	3.414 (1.524–7.648)
rs2981579	CC	No	14	29		
	CC	Yes	13	6	0.009	4.488 (1.409–14.298)
	CT+TT	No	83	67	0.008	2.566 (1.256–5.243)
	CT+TT	Yes	35	21	0.003	3.452 (1.496–7.968)

## Discussion

In the current research, the genetic relationship of *FGFR2* polymorphisms with the risk of osteoporosis occurrence was investigated and revealed in Chinese Han population. First of all, we ensured no significant difference between the case and control groups in age and gender distribution. BMI and alcohol consumption were the influence factors of osteoporosis in this population, but not smoking. AA genotype and A allele of rs2420946 were significantly correlated to the increased risk of osteoporosis occurrence adjusted by age, gender, BMI, cigarette and alcohol consumption. Similarly, the carriage of CC genotype in rs1219648 increased nearly three times risk of osteoporosis development, compared with TT genotype carriers. C allele of rs1219648 might be a risk factor of osteoporosis. Rs2981582 was found to be associated with the risk of osteoporosis in single study of polymorphism, but adjusted by clinical features, the association became not significant. Rs2981579 was not the independent risk factor of osteoporosis. This article is the first time to reveal the role of these four polymorphisms in osteoporosis susceptibility.

Osteoporosis is a complex multi-factor and multi-step disease associated with a mass of factors. So far, multiple associated factors have been identified, including gender, age, nutrition, life style, physical exercise, drug use and disease [19]. Certainly, genetic factors are also indispensable. Vitamin D receptor (*VDR*), Type I collagen (*COL1*), estrogen receptor (*ER*), apolipoprotein E (*ApoE*), bone morphogenetic protein (*BMP*), *LRP5* genes and polymorphisms have been also proved to be involved in osteoporosis etiology [20–22]. FGFs/FGFRs are also reported to be associated with bone information and bone diseases. FGFs include 23 members and FGFRs family consists of FGFR1-5 in humans, a combination of the two can play a role in physiological and pathological processes. FGF2 binding to FGFR1 in the surface of osteoclast can activate downstream MAPK signaling pathway to promote osteoclast differentiation and function exertion [23]. Moreover, FGFR3 also participates in the process which FGF2 regulates osteoclasts through ERK pathway. FGF23 can act on osteoblasts [24] and in animal study of Wei, the increased level and enhanced function of FGF21 in mice obviously cause bone mass loss [25].

However, FGFR2 is rarely studied in osteoporosis. FGFR2 also plays an important role in bone development and bone diseases. It is mostly reported in the occurrence of craniosynostosis syndromes, including Pfeiffer syndrome (PS), Crouzon syndrome (CS) and Apert syndrome (AS) [26]. In skeletal development, FGFR2 regulates the proliferation and differentiation of anterior osteoblasts and osteoblasts located in perichondrium, periosteum and sutura cranii [27]. Moreover, FGFs/FGFR2 has different influence on osteoblasts at different stages of development. *In vitro*, increased FGFR2 level inhibits osteoblasts differentiation and induces apoptosis. FGFR2 protein is encoded by *FGFR2* gene located on chromosome 10q26.13 and the genetic variants of *FGFR2* are the important mechanism of disease. Dong et al. investigated the association of 28 SNPs in FGFR2 with femoral neck (FN) BMD including rs2420946 and rs2981579, only rs11200014 and rs1078806 were detected the significant association with BMD [28]. The results are not strictly consistent with ours, which may derive from different sample size and population from different regions.

In addition, we also revealed the interaction of *FGFR2* four polymorphisms in the present study via linkage disequilibrium analysis and the results showed only one haplotype T-A-C-T was the risk factor of osteoporosis. Although C-A-C-T haplotype contained the risk factors of rs2420946A and rs1219648C, it was not obviously related to the occurrence risk of osteoporosis due to the influence of rs2981582C and rs2981579T. Differently, haplotype T-G-T-C did not carry the risk factors of rs2420946A and rs1219648C, but it had high possibility to increase the occurrence risk of osteoporosis. So the interaction of polymorphisms should be paid attention in the pathogenesis of disease. Besides, the tight interaction of *FGFR2* polymorphisms with drinking status in osteoporosis occurrence risk was also found in the present study.

Some limitations should be stated in the present study. First, the relatively small sample size may limit the statistical power of our study. Based on the minor allele frequencies (MAFs) of the studied polymorphisms in the included patients, we calculated the statistical power of our study using GPower 3.1 software. The results demonstrated that the statistical powers for rs2981582 and rs1219648 polymorphisms were more than 0.6, and the statistical power for analysis of rs2420946 polymorphism was 0.57. Meanwhile, the statistical power for rs2981579 SNP was only 0.34. The relatively low statistical power may reduce the accuracy of our results. Second, in the present study only was included one Chinese Han population, the study population is single. Moreover, the few environmental factors were taken into consideration which may affect the role of polymorphisms in osteoporosis. Therefore, further studied are needed to verify these results and reveal the exact mechanism of *FGFR2* polymorphisms in osteoporosis occurrence with large sample size and multiple populations in different races, considering more environmental factors.

## Conclusion

In conclusion, *FGFR2* rs2420946 and rs1219648 polymorphisms may contribute the risk of osteoporosis occurrence in Chinese Han population, but not rs2981582 or rs2981579. The strong linkage disequilibrium is found among these four polymorphisms and haplotype T-A-C-T may be the risk factor of osteoporosis. The interaction of polymorphisms is also the important hand in osteoporosis pathology. The interaction of *FGFR2* polymorphisms with drinking also plays important roles in osteoporosis development.

## Abbreviations

AGE: agarose gel electrophoresis
BMD: bone mineral density
BMI: body mass index
CI: confidence interval
DXA: dual-energy X-ray absorptiometry
FGFR2: fibroblast growth factor 2 receptor
HWE: Hardy–Weinberg equilibrium
OR: odds ratio
SNP: single nucleotide polymorphism
RTK: receptor tyrosine kinase
